# Population-based study of LAMA monotherapy effectiveness compared with LABA/LAMA as initial treatment for COPD in primary care

**DOI:** 10.1038/s41533-018-0102-x

**Published:** 2018-09-28

**Authors:** Miriam Barrecheguren, Mónica Monteagudo, Marc Miravitlles

**Affiliations:** 1 Pneumology Department, Hospital Universitari Vall d´Hebron. Ciber de Enfermedades Respiratorias (CIBERES), Barcelona, Spain; 2 Primary Care University Research Institute Jordi Gol (IDIAP Jordi Gol), Barcelona, Spain; 3 Universitat Autònoma de Barcelona, Bellaterra (Cerdanyola del Vallès), Spain

## Abstract

This epidemiological study aimed to describe and compare the characteristics and outcomes of COPD patients starting treatment with a long-acting anti-muscarinic (LAMA) or a combination of a long-acting beta-2 agonist (LABA)/LAMA in primary care in Catalonia (Spain) over a one-year period. Data were obtained from the Information System for the Development in Research in Primary Care (SIDIAP), a population database containing information of 5.8 million inhabitants (80% of the population of Catalonia). Patients initiating treatment with a LAMA or LABA/LAMA in 2015 were identified, and information about demographic and clinical characteristics was collected. Then, patients were matched 1:1 for age, sex, FEV1%, history of exacerbations, history of asthma and duration of treatment, and the outcomes between the two groups were compared. During 2015, 5729 individuals with COPD started treatment with a LAMA (69.8%) or LAMA/LABA (30.2%). There were no remarkable differences between groups except for a lower FEV1 and more previous hospital admissions in individuals on LABA/LAMA. The number of tests and referrals was low and decreased in both groups during follow-up. For the same severity status, the evolution was similar with a reduction in exacerbations in both groups. Treatment was changed during follow-up in up to 34.2% of patients in the LABA/LAMA and 26.3% in the LAMA group, but adherence was equally good for both. Our results suggest that initial therapy with LAMA in monotherapy may be adequate in a significant group of mild to moderate patients with COPD and a low risk of exacerbations managed in primary care.

## Chronic lung disease: Simple and effective treatment

A single rather than combined long-acting inhaler therapy may be adequate for most patients when treating mild to moderate chronic lung disease. Marc Miravitlles at the Hospital Universitari Vall d’Hebron, Barcelona, Spain, and co-workers have shown that, in the initial stages of chronic obstructive pulmonary disease (COPD), treatment with an inhaled drug called a long-acting anti-muscarinic agent (LAMA) is as effective as an alternative inhaler that combines LAMA with another drug (LABA). The researchers identified 5729 COPD patients from Catalonia starting on inhaled treatment in 2015 and followed up on their progress after 1 year. Patients starting on LAMA monotherapy were matched closely in terms of demographics and previous medical history to those starting on LAMA/LABA treatment. The team found no remarkable differences in clinical characteristics between the groups over the year.

## Introduction

The aim of pharmacological treatment in chronic obstructive pulmonary disease (COPD) is to reduce symptoms, decrease the frequency and severity of exacerbations and improve exercise tolerance.^1^ Previous studies have demonstrated the efficacy of long-acting bronchodilators (LABD) in improving lung function, respiratory symptoms, exacerbations, exercise capacity and quality of life^2^ and constitute the cornerstone of COPD treatment.^1,3,4^ Among LABD, long-acting anti-muscarinic agents (LAMA) have shown greater efficacy in the prevention of exacerbations compared to long-acting β2-agonists (LABA),^5,6^ and therefore, guidelines recommend the use of LAMA over LABA as the initial treatment in COPD.^1,3,4^

In recent years, new treatments for COPD have been launched, most being LABD, inhaled corticosteroids (ICS) and combinations of the two, with the objective of improving patient outcomes in COPD. Recent studies have suggested that dual treatment with a LAMA and a LABA results in greater bronchodilation and improved symptoms and health status compared to a LABD in monotherapy.^7,8^ However, most studies assessing the efficacy of double bronchodilation compared with monotherapy have included moderate to severe patients with dyspnoea according to the modified Medical Research Council (mMRC) ≥ 2 scale,^2,7,8^ excluding milder patients who may not present a significant improvement with the addition of a second LABD. In addition, there is a lack of follow-up studies in real life in primary care investigating the effectiveness of LABD and its combination with other agents in the usual clinical practice. The use of large population-based databases may help to increase the knowledge on real-life use of bronchodilator treatment in primary care. Therefore, the objective of this study was to describe the characteristics and outcomes of COPD patients starting inhaled treatment with a LAMA or a combination of LABA/LAMA in primary care in Catalonia (Spain) over a 1 year period.

## Results

During 2015, we identified 5729 individuals with a codified diagnosis of COPD who started treatment with either a LAMA (4001 or 69.8%) or LAMA/LABA (1728 or 30.2%). These patients constituted the population of our study. Among the patients receiving a LAMA/LABA, 68.3% received the combination in the same inhalation device.

### Baseline characteristics

In total, 76.9% were men with a mean age of 66.3 (SD 11.1) years, with no differences between the LAMA or LAMA/LABA groups. The patients were diagnosed a mean of 3.3 (4.9) years before inclusion, with significant differences between groups [3.9 (5.3) years for the LABA/LAMA group vs. 3.1 (4.7) years for the LAMA patients, *p* < 0.001]. The most frequent comorbidities were hypertension (57.7%), diabetes mellitus (25.2%), dyslipidemia (50.9%) and depression (18.3%).

Spirometry result with a FEV1 was available in only 40% of the patients. Individuals treated with a combination of two bronchodilators presented more severe disease, with a lower FEV1 % (56.9% (18.5) vs. 65.0% (18.7), *p* < 0.001) and included a greater number of patients classified as GOLD 3 and GOLD 4. Distribution by phenotypes was similar between the two groups. Patients treated with double bronchodilation tended to have more hospital admissions for all causes during the previous year but not for respiratory causes, although numbers were very small. The baseline characteristics of the cohort and by treatment group are presented in Table 1.

**Table 1:** Baseline characteristics of the patients studied

	Total *N* = 5729	Patients starting on LAMA *N* = 4001	Patients starting on LABA/LAMA *N* = 1728	*p*-value
Sex (men)	4406 (76.9)	3043 (76.1)	1363 (78.9)	0.02
Age, years	66.25 (11.1)	66.42 (11.1)	65.84 (11.1)	NS
Years from diagnosis	3.33 (4.9)	3.08 (4.7)	3.91 (5.3)	<0.001
BMI (Kg/m^2^)	29.14 (5.3)	29.04 (5.2)	29.36 (5.4)	NS
Smoking history (*n*,%)				NS
Never smoker	1115 (19.5)	792 (19.8)	323 (18.7)	
Active smoker	2232 (39)	1586 (39.6)	646 (37.4)	
Former smoker	2318 (40.5)	1588 (39.7)	730 (42.2)	
Unknown	64 (1.1)	35 (0.9)	29 (1.7)	
Phenotype (*n*, %)				NS
ACO	183 (3.2)	128 (3.2)	55 (3.2)	
Non exacerbator	1303 (22.7)	890 (22.2)	413 (23.9)	
Exacerbator	4243 (74.1)	2983 (74.6)	1260 (72.9)	
Comorbidities				
Asthma	262 (4.6)	184 (4.6)	78 (4.5)	NS
Bronchiectasis	233 (4.1)	180 (4.5)	53 (3.1)	0.01
OSA	331 (5.8)	232 (5.8)	99 (5.7)	NS
Ischaemic heart disease	808 (14.1)	570 (14.2)	238 (13.8)	NS
Heart failure	483 (8.4)	306 (7.6)	177 (10.2)	0.001
Atrial fibrilation	593 (10.4)	411 (10.3)	182 (10.5)	NS
Hypertension	3307 (57.7)	2303 (57.6)	1004 (58.1)	NS
Diabetes mellitus	1443 (25.2)	1002 (25)	441 (25.5)	NS
Dyslipidemia	2915 (50.9)	2069 (51.7)	846 (49)	0.05
Depression	1049 (18.3)	746 (18.6)	303 (17.5)	NS
Osteoporosis	396 (6.9)	290 (7.2)	106 (6.1)	NS
GERD	299 (5.2)	212 (5.3)	87 (5)	NS
Complementary tests				
Spirometries with FEV1	2271 (39.6)	1584 (39.6)	687 (39.8)	NS
FEV1 %	62.5 (18.9)	65.0 (18.6)	56.9 (18.5)	<0.001
Severity *N* (%) (*n* = 2271)				<0.001
Gold 1	354 (15.6)	296 (18.7)	58 (8.4)	
Gold 2	1415 (62.3)	1013 (64)	402 (58.5)	
Gold 3	424 (18.7)	236 (14.9)	188 (27.4)	
Gold 4	78 (3.4)	39 (2.5)	39 (5.7)	
Exacerbations during the previous 12 months, *n* (%)				NS
0 exacerbations	2685 (46.9)	1871 (46.7)	814 (47.1)	
1 exacerbations	1741 (30.4)	1240 (31.0)	501 (28.9)	
≥2 exacerbations	1303 (22.7)	890 (22.2)	413 (23.9)	
Hospital admission during the previous 12 months, *n* (%)				
Total	449 (7.8)	294 (7.3)	155 (9)	0.03
Respiratory hospital admission	22 (0.4)	13 (0.3)	9 (0.5)	NS

Data are expressed as mean (SD) unless specified otherwise

*BMI* body mass index, *ACO* asthma COPD overlap, *OSA* obstructive sleep apnoea, *GERD* gastrooesophageal reflux disease, *FEV1* forced expiratory volume in 1 s, *GOLD* global initiative for obstructive chronic pulmonary disease

### Longitudinal analysis: matching sub-cohort

**Fig. 1:**
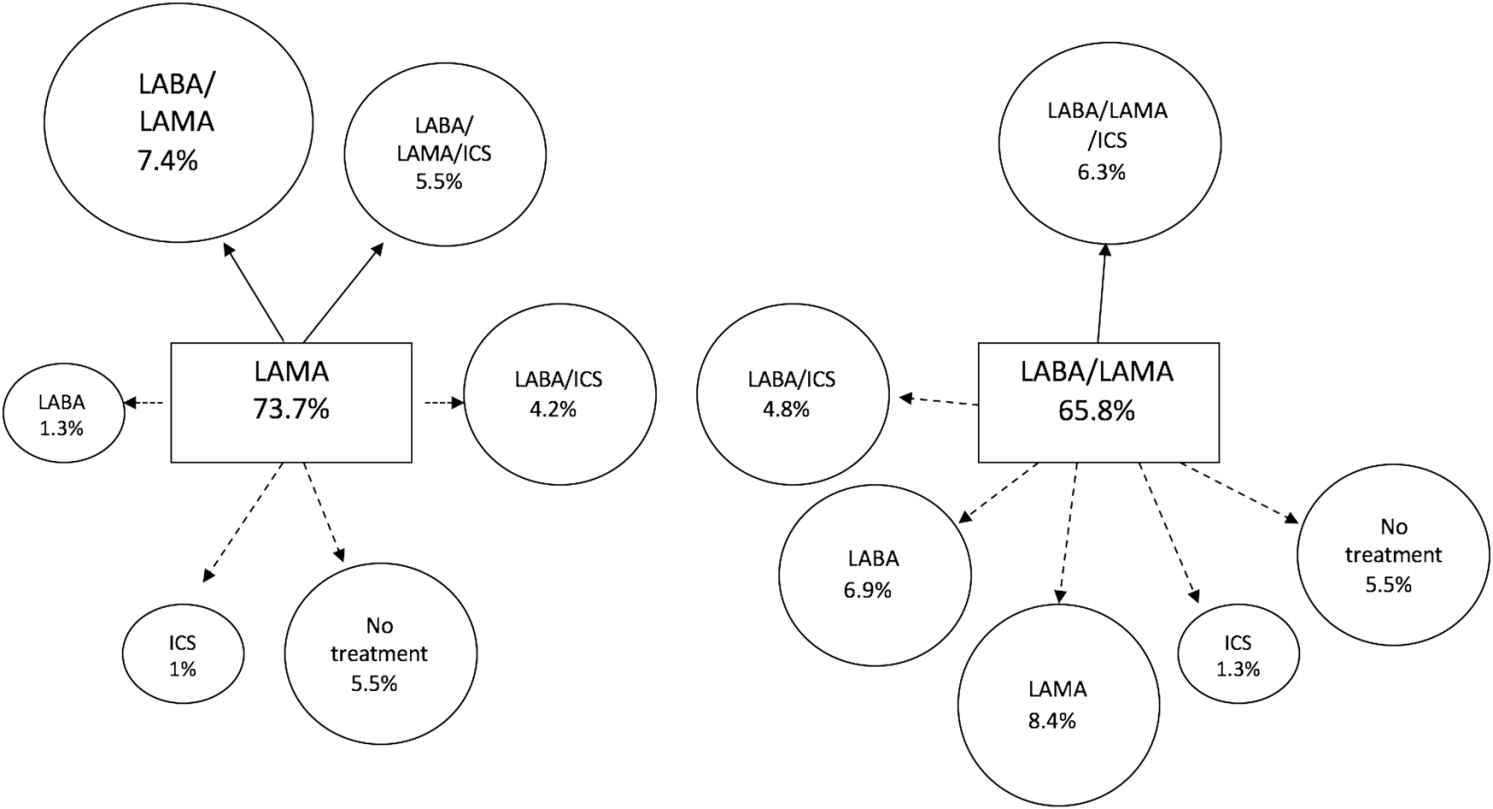
Changes in the treatment pattern at 12 months of follow-up compared to baseline. Continous arrow: step-up or equivalent treatment; dashed arrow: step-down treatment. LABA long-acting beta-2 agonist, LAMA long-acting anti-muscarinic, ICS inhaled corticosteroid

For the longitudinal analysis we identified 1897 (33%) individuals who fulfilled the criteria for the diagnosis of COPD (Fig. 1). Finally, of these, matching was possible for 524 patients from each treatment group.

As expected, there were no differences in the baseline characteristics between groups, although the time from diagnosis to treatment was longer in the combined group (3.7 (5.1) years vs. 2.8 (4.3) years, *p* = 0.002) (Table 2).

**Table 2:** 12-month follow up. Intra- and between groups differences based on initial treatment (LAMA vs. LAMA/LAMA)

	Patients starting on LAMA *N* = 524			Patients starting on LABA/LAMA *N* = 524			
	Baseline	Follow-up	Change % (follow-up-baseline) (CI)	Baseline	Follow-up	Change % (follow-up-baseline) (CI)	Differences between groups (change LAMA—change LABA/LAMA) (CI)
Tests							
Spirometries with recorded FEV_1_	524 (100)	119 (22.7)	−77.3 (−80.9; −73.7)***	524 (100)	132 (25.2)	−74.8 (−78.5; −71.1)***	−2.5 (−7.6; 2.7)
FEV_1,_ %	58.6 (18.8)	60.8 (17.6)	2.1 (0.2; 6)*	58.4 (16.8)	58.6 (16.8)	0.2 (−0.8; 3.4)	1.9 (0.4; 3.9)
Severity *N* (%)							
Gold 1	56 (10.7)	17 (14.3)		42 (8)	8 (6.1)		
Gold 2	325 (62)	73 (61.3)		333 (63.5)	92 (69.7)		
Gold 3	118 (22.5)	26 (21.8)		132 (25.2)	30 (22.7)		
Gold 4	25 (4.8)	3 (2.5)		17 (3.2)	2 (1.5)		
Blood tests	379 (72.3)	359 (68.5)	−3.8 (−9.3; 1.7)	382 (72.9)	371 (70.8)	−2.1 (−7.5; 3.3)	−1.7 (−3.8; 0.3)
CXR	245 (46.8)	90 (17.2)	−29.6 (−34.9; −24.2)***	218 (41.6)	101 (19.3)	−22.3 (−27.7; −16.9)***	−7.3 (−12.5; −2)**
CT scan	45 (8.6)	27 (5.2)	−3.4 (−6.5; −0.4)*	49 (9.4)	23 (4.4)	−5 (−8; −1.9)**	1.5 (−0.9; 4)
Use of healthcare resources							
Patients with at least one exacerbation, *n* (%)	299 (57.1)	226 (43.1)	−13.9 (−19.9; −7.9)***	303 (57.8)	258 (49.2)	−8.6 (−14.6; −2.6)**	−5.3 (−9.2; −1.5)***
No. of exacerbations	1.1 (1.45)	0.85 (1.43)	−0.3 (−0.4; −0.1)***	1.23 (1.63)	1.04 (1.61)	−0.2 (−0.3; −0.1)**	−0.1 (−0.2; 0.1)
Patients with at least one hospital admission, n (%)	30 (5.7)	38 (7.3)	1.5 (−1.5; 4.5)	38 (7.3)	45 (8.6)	1.3 (−1.9; 4.6)	0.2 (−1.2; 1.6)
Hospital admissions	1.53 (0.86)	1.47 (1.01)	−0.4 (−1.9; 1.1)	1.29 (0.65)	1.62 (1.32)	0.8 (−0.6; 2.1)	−1.2 (−3.1; 0.8)
Patients with at least one respiratory hospital admission, n (%)	3 (0.6)	2 (0.4)	−0.2 (−1; 0.6)	1 (0.2)	1 (0.2)	0 (−0.5; 0.5)	−0.2 (−0.6; 0.2)
Respiratory hospital admissions	1.33 (0.58)	1 (0)	–	0	0	–	–
Patients with at least one visit to GP, n (%)	519 (99)	520 (99.2)	0.2 (−0.9; 1.3)	520 (99.2)	522 (99.6)	0.4 (−0.5; 1.3)	−0.2 (−0.8; 0.5)
Visits to GP	8.2 (4.9)	8.2 (5.5)	0 (−0.5; 0.4)	8.6 (5.7)	9.1 (6.6)	0.5 (0; 1)*	−0.5 (−1.2; 0.1)
Patients with at least one nurse visit, *n* (%)	507 (96.8)	475 (90.6)	−6.1 (−9; −3.2)***	500 (95.4)	480 (91.6)	−3.8 (−6.8; −0.8)*	−2.3 (−4.9; 0.3)
Nurse visits	6.8 (9.8)	7.5 (10.8)	0.5 (−0.1; 1)	6.7 (6.8)	7.01 (8.4)	0.2 (−0.5; 0.9)	0.3 (−0.6; 1.2)
Patients with at least one visit to pulmonologist, *n* (%)	1 (0.2)	0	−0.2 (−0.6; 0.2)	1 (0.2)	1 (0.2)	0 (−0.5; 0.5)	−0.2 (−0.6; 0.2)
Visits to pulmonologist	0	0	–	0	0	–	–
Referrals to specialist	109 (20.8)	38 (7.3)	−13.5 (−17.7; −9.4)***	144 (27.5)	33 (6.3)	−21.2 (−25.5; −16.8)***	7.6 (3.1; 12.2)*
Sick leave	27 (5.2)	16 (3.1)	−2.1 (−4.5; 0.3)	32 (6.1)	19 (3.6)	−2.5 (−5.1; 0.1)	0.4 (−1.4; 2.2)
Respiratory sick leave	3 (0.6)	4 (0.8)	0.2 (−0.8; 1.2)	10 (1.9)	9 (1.7)	−0.2 (−1.8; 1.4)	0.4 (−0.1; 0.9)

Baseline data were collected during the 12 months prior to the first prescription of a LAMA or LABA/LAMA. Data are expressed as mean (SD) unless specified otherwise

*CI* confidence interval, *CXR* chest x-ray, *CT* computerised tomography, *GP* general practitioner, *FEV1* forced expiratory volume in 1 s, *GOLD* global initiative for obstructive chronic pulmonary disease

*p < 0.05; **p < 0.01; ***p < 0.001

During the 12-month follow-up, only 22.7% of patients in the LAMA group underwent spirometry. The number of chest X-rays and computerised tomography (CT) lung studies also significantly decreased in the LAMA group (Table 2). This group also showed a significant reduction in the number of patients presenting an exacerbation compared to the previous year (−13.9%, 95% CI: −19.9;−7.9; *p* < 0.001) and in the mean number of exacerbations (−0.3, 95% CI: −0.4; −0.1; *p* < 0.001). The percentage of patients consulting with a nurse or being referred to a specialist also significantly decreased in the LAMA group during the one-year follow-up.

Patients treated with a LABA/LAMA also showed a reduction in the number of spirometries chest X-rays and CT scans performed (Table 2). This group showed a reduction in the percentage of patients presenting an exacerbation (−8.6%, 95% CI: −14.6; −2.6; *p* < 0.001), and in the mean number of exacerbations (−0.2, 95% CI: −0.3; −0.1; *p* < 0.001). In addition, fewer patients consulted a nurse or were referred to a pulmonologist compared to the year before initiating treatment (−21.2%, 95% CI: −25.5; −16.8; *p* < 0.001).

On comparing the two groups, patients treated with a LAMA showed a greater reduction in the number of chest X-rays performed and the number of patients with any exacerbation (percentage difference −5.3%, 95% CI: −9.2; −1.5; *p* < 0.001). Patients receiving LAMA were less frequently referred to the pulmonologist (mean difference 7.6%, 95% CI: 3.1; 12.2; *p* < 0.05).

### Changes in treatment and adherence during follow-up

A total of 439 (83.8%) of the patients initially treated with a LAMA continued receiving this treatment over the 1 year follow-up, although only 73.7% were treated with LAMA alone. The percentage of patients who continued with a LABA/LAMA combination was 65.8% (Table 3).

**Table 3:** Pharmacological treatment at the first prescription of a LAMA or a LABA/LAMA and after a 12-month follow-up

	Patients starting on LAMA			Patients starting on LABA/LAMA			
	*N* = 524			*N* = 524			
	Beginning	End (12 month)	Change %	Beginning	End (12 month)	Change %	Differences between groups (change LAMA—change LABA/LAMA) (CI)
			(End-beginning)(CI)			(End-beginning) (CI)	
Treatment by drug							
SABA	92 (17.6)	68 (13)	−4.6 (−8.9; −0.2)*	98 (18.7)	77 (14.7)	−4 (−8.5; 0.5)	−0.6 (−3; 1.9)
SAMA	47 (9)	34 (6.5)	−2.5 (−5.7; 0.7)	54 (10.3)	38 (7.3)	−3.1 (−6.5; 0.4)	0.6 (−1.4; 2.6)
LABA	0	27 (5.2)	5.2 (3.3; 7)***	196 (37.4)	117 (22.3)	−15.1 (−20.5; −9.6)***	20.2 (16.6; 23.8)***
LAMA	524 (100)	439 (83.8)	−16.2 (−19.4; −13.1)***	184 (35.1)	140 (26.7)	−8.4 (−14; −2.8)**	−7.8 (−11.8; −3.9)***
SABA + SAMA	0	0	0 (0; 0)	0	1 (0.2)	0.2 (−0.2; 0.6)	−0.2 (−0.6; 0.2)
LABA + LAMA	0	22 (4.2)	4.2 (2.5; 5.9)***	343 (65.5)	282 (53.8)	−11.6 (−17.5; −5.7)***	15.8 (12.6; 19.1)***
LABA + ICS	0	49 (9.4)	9.4 (6.9; 11.8)***	0	41 (7.8)	7.8 (5.5; 10.1)***	1.5 (−1.9; 4.9)
ICS	0	14 (2.7)	2.7 (1.3; 4.1)***	0	25 (4.8)	4.8 (2.9; 6.6)***	−2.1 (−4.4; 0.2)
Methylxanthines	1 (0.2)	1 (0.2)	0 (−0.5; 0.5)	2 (0.4)	1 (0.2)	−0.2 (−0.8; 0.5)	0.2 (−0.2; 0.6)
IPDE4	0	0	0 (0; 0)	1 (0.2)	1 (0.2)	0 (−0.5; 0.5)	0 (0; 0)

*SABA* short-acting beta-2 agonist, *SAMA* short-acting anti-muscarinic, *LABA* long-acting beta-2 agonist, *LAMA* long-acting anti-muscarinic, *ICS* inhaled corticosteroid, *PDE4* phosphodiesterase 4 inhibitor

*p < 0.05; **p < 0.01; ***p < 0.001

We observed good adherence to treatment (proportion of days covered [PDC] > 80%) in 71% of patients initiating with a LAMA and 66.6% for LABA/LAMA. In the LABA/LAMA group, adherence was similar for patients treated with one or two devices (67.1 and 65.7%, respectively).

Regarding changes in treatment, in the LAMA group, treatment was escalated in 11.6% during the year of follow-up (7.4% to LABA/LAMA and 5.5% to triple therapy), 4.2% were switched to LABA/ICS and in 5.5% the treatment was de-escalated to no inhaled treatment.

In the LABA/LAMA group, treatment was escalated to triple therapy in 6.3%, being de-escalated to LABD in monotherapy in 15.3% (8.4% LAMA and 6.9% LABA), 4.8% changed to LABA/ICS and 6.5% to no inhaled therapy (Table 4) (Fig. 1).

**Table 4:** Changes in treatment pattern according to severity (GOLD stages) during the 12-month follow-up in patients in both groups matched for age, sex, FEV1%, previous history of exacerbations, history of asthma and duration of treatment

	Patients starting on LAMA *N* = 524					Patients starting on LABA/LAMA *N* = 524				
	Total	Gold 1 *N* = 56	Gold 2 *N* = 325	Gold 3 *N* = 118	Gold 4 *N* = 25	Total	Gold 1 *N* = 42	Gold 2 *N* = 333	Gold 3 *N* = 132	Gold 4 *N* = 17
LAMA	386 (73.7)	47 (83.9)	245 (75.4)	78 (66.1)	16 (64)	44 (8.4)	6 (14.3)	31 (9.3)	7 (5.3)	0
LABA	7 (1.3)	0	5 (1.5)	2 (1.7)	0	36 (6.9)	1 (2.4)	30 (9)	5 (3.8)	0
ICS	5 (1)	1 (1.8)	2 (0.6)	1 (0.8)	1 (4)	7 (1.3)	1 (2.4)	4 (1.2)	2 (1.5)	0
LABA/LAMA	39 (7.4)	3 (5.4)	20 (6.2)	16 (13.6)	0	345 (65.8)	28 (66.7)	209 (62.8)	95 (72)	13 (76.5)
LABA/ICS	22 (4.2)	1 (1.8)	12 (3.7)	5 (4.2)	4 (16)	25 (4.8)	1 (2.4)	13 (3.9)	8 (6.1)	3 (17.6)
LAMA/ICS	7 (1.3)	0	4 (1.2)	3 (2.5)	0	0	0	0	0	0
LAMA/LABA/ICS	29 (5.5)	1 (1.8)	17 (5.2)	8 (6.8)	3 (12)	33 (6.3)	2 (4.8)	20 (6)	10 (7.6)	1 (5.9)
No treatment	29 (5.5)	3 (5.4)	20 (6.2)	5 (4.2)	1 (4)	34 (6.5)	3 (7.1)	26 (7.8)	5 (3.8)	0

Data are expressed as *n* (%)

*LABA* long-acting beta-2 agonist, *LAMA* long-acting anti-muscarinic, *ICS* inhaled corticosteroid

Changes in treatment differed according to severity. In the LAMA group less severe patients (GOLD 1 and 2) tended to continue on a LAMA (83.9% of GOLD 1 and 75.4% of GOLD 2). The most frequent change of treatment was escalation to LABA/LAMA or discontinuation of all treatment. In the LABA/LAMA group, only two thirds of GOLD 1 and 2 patients continued with the same treatment. Patients were frequently de-escalated to one LABD (23.7% of GOLD 1 and 26.1% of GOLD 2) or no maintenance therapy (Table 4).

Up to 13.5% of GOLD 3 patients in the LAMA group were escalated to LABA/LAMA, while GOLD 4 were more frequently escalated to triple therapy or were switched to LABA/ICS. In the LABA/LAMA group, GOLD 4 patients were switched to triple therapy (5.9%) and more frequently to LABA/ICS (17.6%).

## Discussion

The results of this study show that COPD patients starting treatment with a LAMA or a LABA/LAMA in the primary care setting had no remarkable differences in clinical characteristics except in regard to disease severity; patients initially treated with the combination of bronchodilators had a lower FEV1 and a greater number of previous hospital admissions. The number of diagnostic tests and referrals was low in the two groups and decreased during the one-year follow up, especially in those receiving a LAMA. After performing a matching analysis, we observed that for the same severity status, the evolution was similar for individuals treated with a LAMA or LABA/LAMA. In up to 34.2% of patients in the LABA/LAMA group and 26.3% in the LAMA group a change was made in treatment during follow-up, but the adherence was equally good in both treatment schedules.

During the year of the study, 5729 COPD individuals started treatment with either a LAMA or a LABA/LAMA. Among them, 1897 had confirmed COPD (coded for COPD plus airflow obstruction and smoking history). In Catalonia, a region with approximately 7.5 million inhabitants, a recent study also carried out using the SIDIAP (Information System for the Development of Research in Primary Care) database^9^ found that approximately 7000 individuals were diagnosed with COPD every year between 2007 and 2012, but only 11.3 and 3.7% were initially treated with a LAMA or a LABA/LAMA combination in 2012, respectively, suggesting that the use of LABD has increased in recent years. Interestingly, almost two thirds of the patients started with a LAMA and only one third with a LABA/LAMA combination, which is consistent with data from the UK where the most frequent initial treatment for COPD in primary care is a LAMA.^10^

In our study, only 40% of the patients had undergone spirometry with an available FEV1 measurement, which is consistent with previous data from primary care in our country^9,11,12^ and in other European countries.^13–15^ Moreover, along the 12 months after the initiation of the therapy, a follow-up spirometry was performed in only one quarter of the patients in each group.

We found that patients with a codified diagnosis of COPD treated with the combination of bronchodilators had a lower FEV1 and more previous hospital admissions for all causes. However, we found no differences in other relevant clinical characteristics, such as phenotype or the number of previous out-patient exacerbations. Furthermore, previous hospital admissions for respiratory causes were similar, and very low, in both groups. We observed the same results when we analyzed only patients with confirmed COPD (data not shown).

To avoid biases due to different degree of severity, we performed a matching analysis to compare the evolution of COPD patients by group therapy. Firstly, in order to exclude asthma or other respiratory diseases possibly miscodified as COPD as far as possible, we selected only patients who had a history of smoking and a FEV1/FVC < 0.7. Secondly, we matched individuals from both treatment groups by age, sex, FEV1, previous history of exacerbations, history of asthma and duration of treatment during the first 12 months of follow-up. After matching, we found that the evolution of patients treated with a LAMA or a LABA/LAMA did not significantly differ. The assessments performed during follow-up were similar for both groups with a low number of tests performed, visits to the nurse and referrals, although chest X-rays and referrals to the chest physician were even less frequent in the LAMA group.

Patients in both groups presented a significant reduction in the number of exacerbations after the initiation of treatment, consistent with previous clinical trials.^8,16^ Interestingly, in the LAMA group there was a greater reduction in the number of patients presenting an exacerbation, compared to patients initially receiving two bronchodilators. In contrast with these results, previous randomised control trials have shown that a LABA/LAMA combination can improve exacerbation outcomes in comparison to monotherapy. The Spark study,^16^ which was designed to evaluate the effect of dual bronchodilator treatment on exacerbations, reported a reduced annual rate of moderate or severe exacerbations with indacaterol/glycopyrronium compared to glycopyrronium alone. These differences in outcomes are probably due to the population studied. Spark included severe COPD individuals with a FEV1 < 50% and at least one exacerbation in the previous year, while our study population included less severe patients with a majority of GOLD 2 and 45% of patients without any exacerbation in the previous year.

Changes in treatment were more frequent in the LABA/LAMA group. The most frequent change was de-escalation to a LABA or a LAMA (15.3%) followed by no treatment. This was more common in milder patients, while GOLD 4 patients tended to continue with the combination of bronchodilators. In comparison, changes in treatment were slightly less frequent in the LAMA group, with the most frequent change being escalation to a LABA/LAMA combination or triple therapy (12.9%), while 6.8% switched to a LABA or LABA/ICS. An analysis of the Pharmo database in the Netherlands found more frequent changes in treatment in patients on LAMAs, with persistence rates at 1, 2, and 3 years of only 25%, 14% and 8% respectively.^17^ In contrast with our data, most patients were changed to ICS/LABA. Wurst et al.^18^ also observed a lower persistence to newly initiated LAMA, with 10% of patients receiving the addition of other therapies or 9% being switched from LAMA mainly to LABA/ICS and with many patients discontinuing therapy. Another study that described the changes to triple therapy found that starting with a LAMA was one of the least frequent schedules and represented only 9.5% among all the patients finally receiving triple therapy.^19^

In our population, adherence was good and did not significantly differ between the LAMA (71%) and LABA/LAMA groups (66.6%). Other studies analysing the persistence of treatment with LAMAs in Spain have described similar results. One study only included individuals treated with aclidinium or tiotropium and observed an initially high persistence with LAMA that progressively decreased to 50% after 1 year of follow-up.^20^ Izquierdo et al.^21^ conducted another population-based study in Castile-La Mancha (Spain) and observed a high rate of compliance (from 80 to 120%) in patients treated with LAMAs.

The main limitation of the present study is the lack of data on dyspnoea (mMRC) or the COPD assessment test, which may be implicated in therapeutic decision making. However, an observational study aimed at identifying predictors of physician treatment choice in primary care in the UK observed that the mMRC score was a weak predictor of therapeutic choice,^22^ and had no impact on the treatment modifications to triple therapy.^19^ Other limitations inherent to database studies are diagnostic and miscoding biases. To minimise these biases we only included individuals with confirmed COPD (codified diagnosis plus smoking history and FEV1/FVC < 0.7) in the longitudinal matched analysis. In contrast, the main strength of our study is the large coverage of the database, including more than 80% of the population of Catalonia (Spain), thereby ensuring the representativeness of our data.

In conclusion, our study suggests that Primary Care physicians in Catalonia more frequently prescribe a LABA/LAMA combination instead of a LAMA for the most severe COPD patients, consistent with guideline recommendations.^1,3,4,23^ Most patients initiating treatment with a LAMA remain stable during follow-up and frequently continue on the same treatment over time. In contrast, patients initially treated with a LABA/LAMA combination are more likely to be switched to other treatment schedules, particularly de-escalation to a single LABD. This was more frequently observed in GOLD 1 and 2 patients, which suggests unnecessary overtreatment in milder stages of the disease. Adherence to treatment was high in both treatment groups. Our results suggest that initial therapy with LAMA in monotherapy may be adequate for a significant group of mild to moderate patients with COPD and a low risk of exacerbations managed in primary care.^24^

## Methods

This was an epidemiological study with retrospective analysis of longitudinal follow-up data that aimed to compare the characteistics of the COPD patients initiating treatment with a LAMA or a combination of LAMA/LABA in primary care in 2015 and their clinical evolution over a 12-month follow-up period.

The data for this study was obtained from the SIDIAP database, a computerised database containing anonymized patient records for the 5.8 million people registered in one of the 279 primary care centres of the Catalan Health Institute (approximately 80% of the population of Catalonia).^25^ All the general practitioners in the Catalan Public Health Service use the same eCAP software to record the clinical information of their patients. Health professionals gather this information using ICD-10 codes and structured forms designed for the collection of variables such as smoking history or body mass index or test results such as spirometries. SIDIAP combines information from the electronic medical records with data from other databases and registers including the Pharmacy Register (medication dispensed in pharmacies) and the National Death registry. The study was approved by the Research and Ethics Committee of the IDIAP Jordi Gol Institute of Research In Primary Care (Barcelona, Spain).

### Study population

For the first objective of the comparison of characteristics of COPD patients initiating treatment with a LAMA or LAMA/LABA, we selected all individuals older than 40 years who started treatment with either a LAMA or a combination of a LAMA and a LABA (separately or in one device) during 2015 and were diagnosed with COPD at or before the date of the first prescription of maintenance therapy. Patients were required to have at least 2 years of continuous data (1 year before and 1 year after the first prescription of maintenance therapy).

**Fig. 2:**
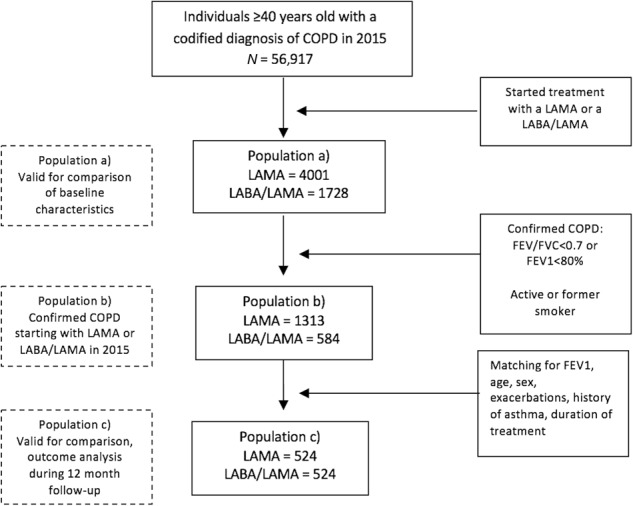
Flowchart of the patients included in the study. SIDIAP information system for research in primary care, COPD chronic obstructive pulmonary disease, LABA long-acting beta-2 agonist, LAMA long-acting anti-muscarinic, Hx history, FEV1 forced expiratory volume in 1 s, FVC forced vital capacity

For the second objective, comparison of the clinical evolution and changes in treatment over a 12-month follow-up period in patients initially treated with a LAMA or a LABA/LAMA, only patients with a confirmed diagnosis of COPD defined as a history of smoking and spirometry with FEV1/FVC < 0.7 were included, as in previous studies.^9,21^ In addition, patients were matched 1:1 for age, sex, FEV1%, previous history of exacerbations, history of asthma and duration of treatment in order to account for the possible differences in clinical characteristics and the severity of patients initiating treatment with a LAMA in monotherapy or with two bronchodilators (Fig. 2).

### Measurements

Severity was assessed based on GOLD stages in the patients for whom a FEV1 value was available (stage 1 mild, FEV ≥ 80% predicted, stage 2 moderate 50% ≤ FEV1 < 80% predicted; stage 3 severe, 30% ≤ FEV1 < 50% predicted; stage 4 very severe FEV < 30% predicted).^1^ Exacerbations were identified by diagnostic codes and by treatment (patients receiving antibiotics and/or oral corticosteroids in the absence of another codified infectious event such as tonsillitis or urine infection).

Patients with two or more exacerbations during the year before initiation of therapy were classified as having a frequent exacerbator phenotype, while those with a previous history of asthma were included in the asthma COPD overlap (ACO) phenotype, and the remaining COPD patients were considered non exacerbators.

A change in treatment was defined as the registration of a new LABD or ICS for at least 60 days and after 2 months following the initiation of treatment with or without new billing of the treatment of interest.

### Statistical analysis

A descriptive analysis of the initial COPD population identified by a diagnostic code was performed. For qualitative variables, absolute frequencies and corresponding percentages were calculated. Quantitative variables with a normal distribution were described by mean and standard deviation, while those that did not follow a normal distribution were described using the median and 25–75 percentiles.

Categorical variables were compared using the Chi-square or Fisher exact test when applicable. Quantitative variables were compared using the *T*-test or Mann–Whitney *U*.

For the follow-up study, the two study sub cohorts were constructed based on a 1:1 matching using the greedy-matching algorithm (without replacement) and the propensity score. We calculated the mean or percentage differences for the different variables between the 12-month follow-up after the first prescription of maintenance therapy and the year before this prescription for both groups independently: those initiating with a LAMA or a LABA/LAMA, followed by calculation of the mean or percentage differences between the two groups.

A logistic regression model was used to establish the propensity score to predict the probability of a patient being treated with a LAMA or LABA/LAMA.

The PDC was used to study therapeutic adherence, A patient was considered to be adherent to treatment when the relation between the proportion of the billed doses of pharmacy and the number of days covered according to the labelling of the product (or PDC) was greater than 0.80.

The matching analyses were performed using the statistical software package Stata/SE version 14 for Windows (Stata Corp. LP, College Station, Texas, US), and SPSS version 22 (SPSS Inc., Chicago, IL, USA) was used for the remaining statistical analyses.

## Data Availability

The data that support the findings of this study are available from IDIAP Jordi Gol but restrictions apply to the availability of these data, which were used under license for the current study, and so are not publicly available. Data are however available from the authors upon reasonable request and with permission of IDIAP Jordi Gol.
